# The CNN model aided the study of the clinical value hidden in the implant images

**DOI:** 10.1002/acm2.14141

**Published:** 2023-09-01

**Authors:** Xinxu Huang, Xingyu Chen, Xinnan Zhong, Taoran Tian

**Affiliations:** ^1^ State Key Laboratory of Oral Diseases National Clinical Research Center for Oral Diseases West China Hospital of Stomatology Sichuan University Chengdu China

**Keywords:** artificial intelligence, convolutional neural network, deep learning, implantation, implants, oral implantology

## Abstract

**Purpose:**

This article aims to construct a new method to evaluate radiographic image identification results based on artificial intelligence, which can complement the limited vision of researchers when studying the effect of various factors on clinical implantation outcomes.

**Methods:**

We constructed a convolutional neural network (CNN) model using the clinical implant radiographic images. Moreover, we used gradient‐weighted class activation mapping (Grad‐CAM) to obtain thermal maps to present identification differences before performing statistical analyses. Subsequently, to verify whether these differences presented by the Grad‐CAM algorithm would be of value to clinical practices, we measured the bone thickness around the identified sites. Finally, we analyzed the influence of the implant type on the implantation according to the measurement results.

**Results:**

The thermal maps showed that the sites with significant differences between Straumann BL and Bicon implants as identified by the CNN model were mainly the thread and neck area. (2) The heights of the mesial, distal, buccal, and lingual bone of the Bicon implant post‐op were greater than those of Straumann BL (*P* < 0.05). (3) Between the first and second stages of surgery, the amount of bone thickness variation at the buccal and lingual sides of the Bicon implant platform was greater than that of the Straumann BL implant (*P* < 0.05).

**Conclusion:**

According to the results of this study, we found that the identified‐neck‐area of the Bicon implant was placed deeper than the Straumann BL implant, and there was more bone resorption on the buccal and lingual sides at the Bicon implant platform between the first and second stages of surgery. In summary, this study proves that using the CNN classification model can identify differences that complement our limited vision.

## 1 INTRODUCTION

Oral implantation and restoration have gradually become the preferred options for restoring missing teeth.^1^ Thus, the increase in their applications has promoted research on the impact of various factors affecting clinical implantation outcomes, as many factors may influence the path of implantology treatment. Pre‐treatment, surgical, and restorative factors could potentially alter the outcome of implant‐based restoration.^2^ Identifying factors and analyzing their impact on treatment outcomes is a major area of concern in implantology.^3^ Those factors are established and scrutinized by health providers based on their clinical information in various forms and medical knowledge and expertise. However, the available medical knowledge and expertise currently established in the field might have limited the vision of health providers during the procedure. Hence, the non‐human assistant identification of clinical factors, unbiased by limited vision, would be an excellent supplement to the wisdom of clinical practitioners.

Artificial intelligence (AI) in various forms is pushing the frontiers of medicine. The idea of deep learning is also being applied to various fields of medicine.^4^, ^5^ Specifically, convolutional neural networks (CNN) are being widely applied. As one of the core models of artificial neural networks and deep learning, CNN is a special deep feedforward network consisting mainly of the input, convolutional, pooling, fully connected, and output layers, which provides computer vision capabilities, including medical image classification.^6^ In the status quo, CNN models have shown accuracy and reliability in medical image classification.^7^, ^8^, ^9^, ^10^, ^11^ However, compared to the well‐established classification, little attention is given to the training process of CNN models. As a non‐human aid to identification, the training process of a neural network model comprises the accumulation of differences in source images.^12^ The training algorithm dictates that the training process naturally identifies and accumulates various factors related to the differences among the images. Particularly, neural network models can extract image differences that humans may never notice.^13^ These differences can be used as entry points for researchers to study the impact of various factors on clinical outcomes. It is notable that the training process of unsupervised networks is processed as a black box and is not disturbed by subjective human factors.^14^ In other words, the CNN training process is based only on feature extraction learning from a large amount of medical data, without the need to label and segment images or classify features before training by people, which is an excellent way to avoid model classification errors due to subjective factors or limitations in the expertise of researchers or clinicians.^8^, ^15^, ^16^ Therefore, CNN has the potential to provide us with an additional complement to study the impact of various clinical factors on implantation outcomes without the bias of limited vision, broadening the horizons of researchers' knowledge in the field of implantology.

Based on the broad application of the classification CNN model, we propose a new and essential application concept of neural networks for evaluating radiographic image recognition results. In other words, we used the CNN model and the gradient‐weighted class activation mapping (Grad‐CAM) to present the differences in implant image identification, which can supplement the limited field of vision of experts and clinicians when studying the influence of various factors on implant images and clinical implantation results. Further reasonable inferences, assumptions, and verifications are made for the identification differences, to help us extract meaningful guidance for clinical implantation work. It is well known that a large amount of imaging data is generated in the clinical work of oral implantation. At present, image data are more commonly used in diagnosing and treating a single patient's disease, and we think that they have not been fully applied. Therefore, we propose a new method using the CNN model that can simultaneously analyze a large number of clinical implantable images and present the identification differences between the images, enabling further application of implant images. After deep learning based on large amounts of data, such differences presented at the overall level can provide reliable direction and inspiration for clinicians, so that they can further study various factors affecting the clinical implant repair effect.The proposed method would reveal novel perspectives on how AI can help clinicians and other medical experts.

## 2 MATERIALS AND METHODS

### 2.1 Collection and processing of image data sets

#### 2.1.1 Data collection

This study was conducted at the Department of Implantology, West China Hospital of Stomatology, Sichuan University. As this was a non‐interventional retrospective trial and all data were analyzed anonymously during the study, no informed consent was required from the participants. Cone beam computed tomography (CBCT) and periapical radiographic images of patients who underwent oral implant treatment at the hospital were acquired between November 2014 and May 2022. There were 1390 periapical radiographs, including 794 training, 389 validation, and 207 test sets. The following two implant systems were included in the collected data sets: Straumann BoneLevel (BL) and Bicon. All radiographic images and CBCT images were attended by the same dentist.

#### 2.1.2 Image processing

Usually, when using a CNN model for learning, the radiographic images used as the dataset should focus on each implant. Therefore, we clipped periapical radiographic images before training to ensure that there was only one target implant in each image. The radiographic images were all at the proximal and distal midplanes of the implant. Subsequently, all manually cropped images were inserted into the computer picture cropping program so that the size of each image was 250 × 250 pixels,^15^ which ensures that the inserted images can allow the CNN to learn and yield accurate results.

### 2.2 Constructing a classification CNN model based on VGG‐16 and transfer learning for deep learning

**FIGURE 1:**
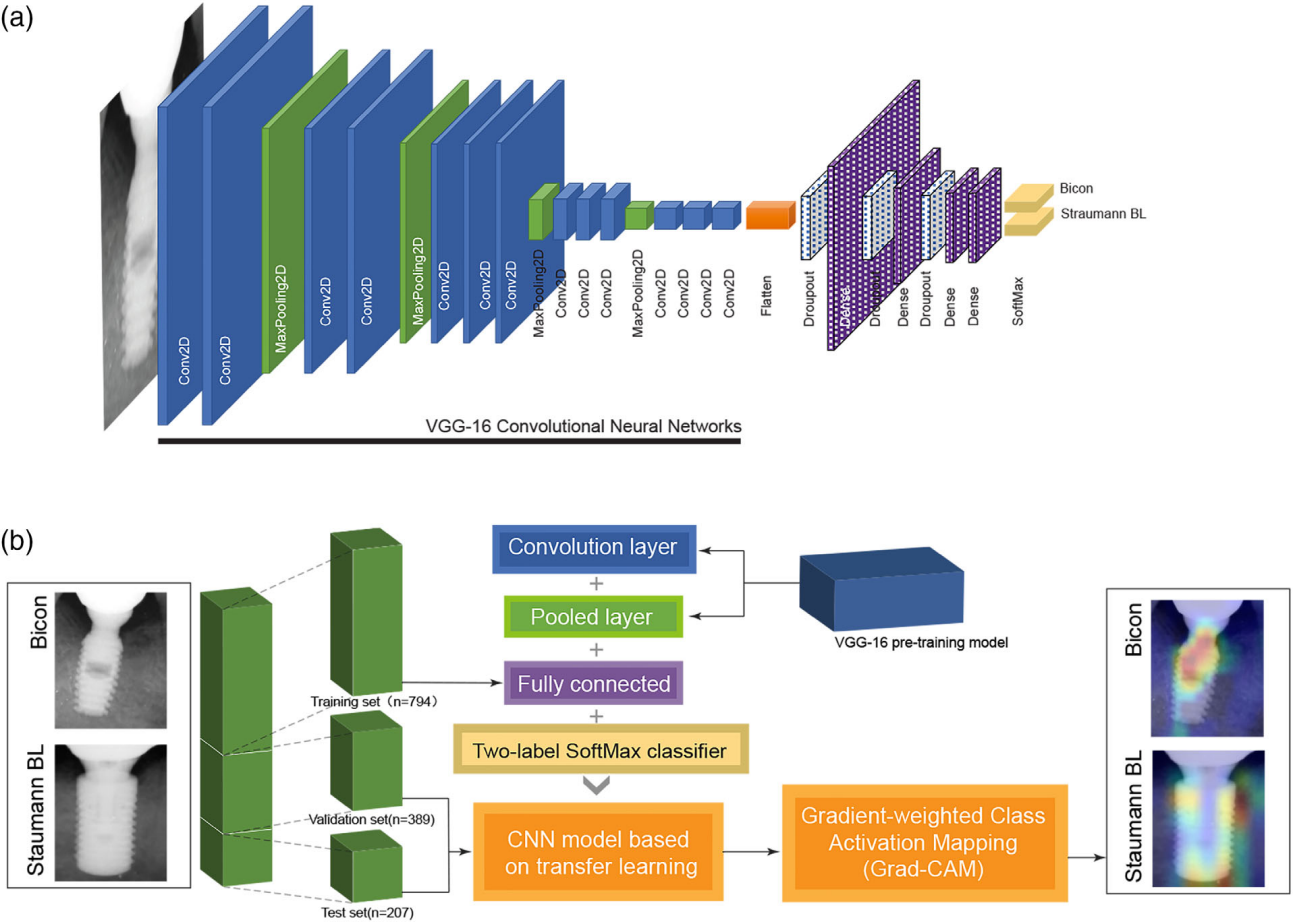
Overview of the general scheme for presenting implant image differences based on CNN models and Grad‐CAM. Part a describes the detailed structure of the CNN model based on VGG‐16, and part b represents the overall process of transfer learning and Grad‐CAM.

We constructed our CNN model based on VGG‐16 for deep learning. VGG‐16 is a CNN model developed by the Visual Geometry Group of Oxford University and Google DeepMind^18^ and used mainly for image identification and classification.^19^ We adopted the convolutional layer structure of the VGG‐16 as the convolutional layer of our CNN model. Furthermore, our model added a dropout layer after the fully connected layer to reduce data overfitting^20^ and a flattened layer to realize the transition from the convolutional layer to the fully connected layer.^7^, ^21^ The structure of the CNN model is illustrated in Figure 1a, and the specific parameters are shown in Table 1. The deep‐learning process of the CNN in this study also adopted the RMSprop optimization algorithm. RMSprop optimizer is based on an accelerating gradient descent speed suitable for CNNs, which can further improve the accuracy and recall rate of the model for implant radiographic image classification.^22^

**TABLE 1:** Structural parameters of the CNN model.

Layers (categories)	Output shape	Number of parameters
Vgg‐16 (functional)	(None, 7, 7, 512)	14714688
Flatten (flatten)	(None, 25088)	0
Dropout (dropout)	(None, 25088)	0
Dense (dense)	(None, 1024)	25691136
Dropout_1 (dropout)	(None, 1024)	0
Dense_1 (dense)	(None, 512)	524800
Dropout_2 (dropout)	(None, 512)	0
Dense_2 (dense)	(None, 256)	131328
Dense_3 (dense)	(None, 2)	514

The training sets required by CNN are enormous, and more than one million training sets are required to complete the training on ImageNet. However, collecting radiographic images of oral implants in such sufficient numbers is difficult. Therefore, to ensure the effectiveness and reliability of deep learning, we adopted transfer learning^23^ and used only the implant datasets to train the fully connected layers. In contrast, the convolutional layer and pooling layer before the fully connected layer were constructed by directly loading the pre‐trained VGG‐16 model to form a complete CNN model for image classification.^9^, ^24^ The transfer learning process used to construct a CNN model to recognize implant radiographic images is shown in Figure 1b.

### 2.3 Presenting thermal maps through Grad‐CAM

Grad‐CAM is a CNN visualization method used to show differences between image categories by generating thermal maps.^23^ In this study, Grad‐CAM was used to visually present the identified sites of the implant, which helped to improve the understanding of the CNN training process.^26^, ^27^ Figure 1b shows the process of visualizing the recognition results of implant radiographic images using Grad‐CAM.

The Grad‐CAM thermal maps were obtained by using the 207 periapical radiographic images from the test set in the CNN model, including 55 Bicon implants and 152 Straumann BL implants. We subsequently divided implants into four sites: apical, body (thread), platform transfer department, and repair parts, including the abutment and screws,^28^ and counted the number of identified sites of the two types of implants obtained from the Grad‐CAM thermal maps. The results were averaged over three counts by a researcher. The percentage of identified sites was obtained by dividing the number at each site by the total number of implants. Statistical analysis was performed using SPSS version 25.0 (IBM Corporation, Armonk, NY, USA), and the chi‐squared test was performed for the counts of the identified sites of the implants.

### 2.4 Measuring bone thickness around the implant

#### 2.4.1 Measurement object

We randomly selected the CBCT images of 77 patients after the first and second stages of surgery from the collected data sets as measurement objects: We collected the images of 77 patients with 59 Bicon implants and 42 Straumann BL implants. We included implants located in the premolar and molar positions.^29^ The tooth positions of the included implants are shown in Table 2, and there was no significant difference between implants (*P* > 0.05).

**TABLE 2:** Tooth positions of the included implants.

	Premolars	Molars
Bicon	8	51
Straumann BL	7	35

#### 2.4.2 Measurement method

We used the distance measurement tool in Onevolume Viewer software to measure each measuring position.

#### 2.4.3 Measurement position

The measurement positions are as follows: bone height on the buccal (H1), lingual (H2), mesial (H3), and distal (H4) sides above the implant platform; The bone thickness on the buccal and lingual aspects of the implant platform (0 mm); bone thickness of the buccal side at 1, 2, 3, and 4 mm under the implant platform; and the bone thickness on the lingual aspect was 1 and 2 mm under the implant platform (Figure 3a).^30^

#### 2.4.4 Statistical analysis

SPSS version 25.0 was used for the statistical analyses. Results are expressed as mean ± standard deviation (SD). After the normality and homogeneity of variance tests, a completely random design *t*‐test was used if the conditions were met. Otherwise, the rank sum test was used. *P* < 0.05 was considered to be statistically significant.

## 3 RESULTS AND DISCUSSION

### 3.1 Differences between the radiographic images of two types of implant identified by the classification CNN model

**FIGURE 2:**
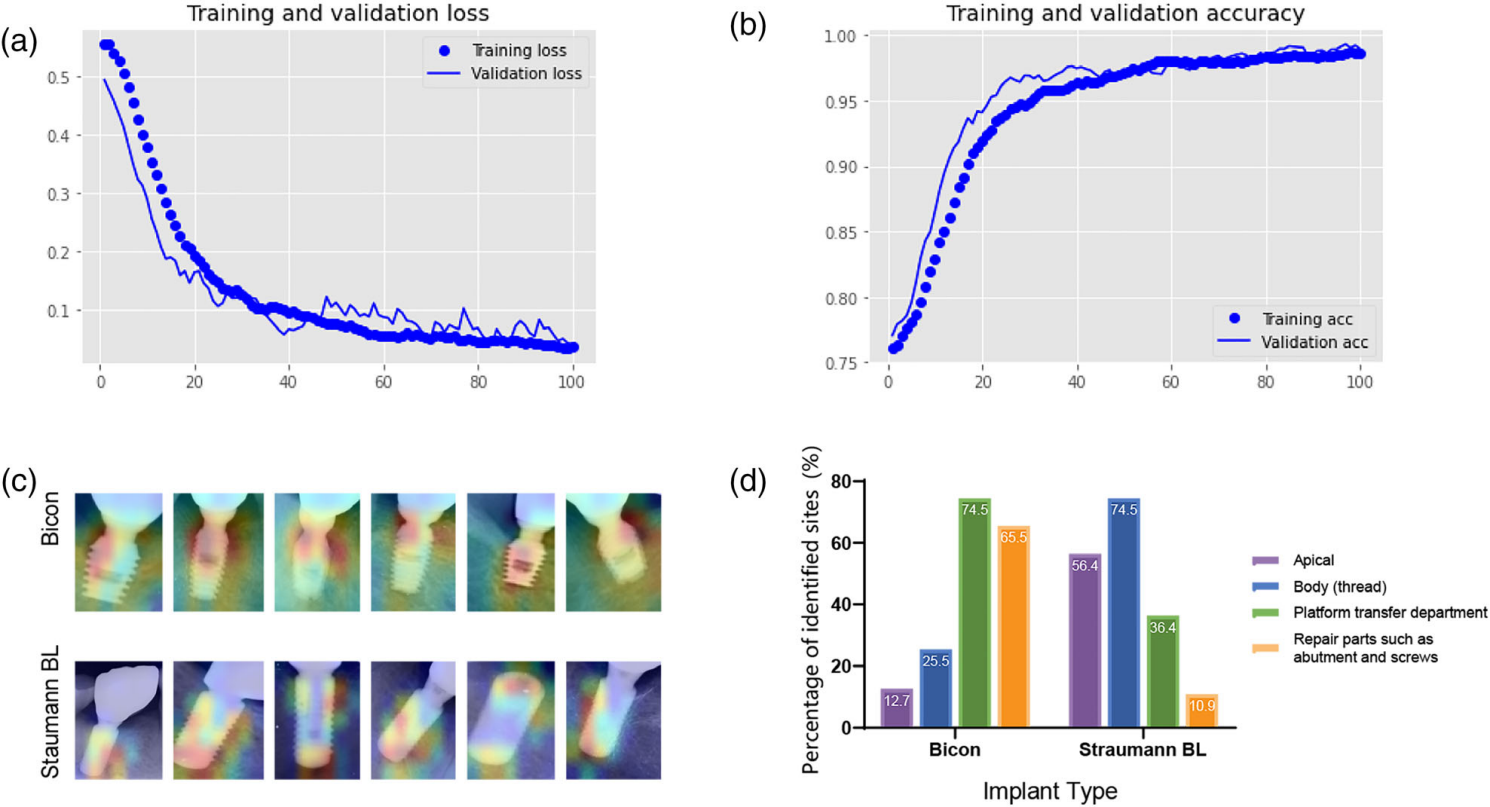
Results of implant image dataset recognized by classification CNN model. Part a is training loss function, and part b describes the classification accuracy. Part c shows part of the thermal maps presented by Grad‐CAM, and part d indicates the statistical results of the implants identification sites.

The classification effect of the CNN model after transfer learning on the two types of implants is shown in Figure 2a and b. According to the output data, the training loss of this model for 207 test sets was only 0.37%, and the classification accuracy rate reached 100.00% for the test dataset. The classification accuracy of CNN for implant radiographic images has been fully confirmed. The results ensure that both the identified differences presented by Grad‐CAM and the analysis based on these differences are reliable.

Part of the deep learning results of the two implants activated by Grad‐CAM is shown in Figure 2c. After classifying the results obtained on 207 thermal maps, we found that there were significant differences in the process of recognition and accumulation of differences in implant radiographic images by the CNN model, and the chi‐square test showed that the difference was statistically significant (*P* < 0.05). As shown in Figure 2d, the sites identified by the CNN of the Bicon implants were mainly the neck area, that is, the platform transfer department and repair parts, including the abutment and screws. The identified sites of the Straumann BL implants are mainly the thread and apex.

### 3.2 Analysis of the reasons behind the differences between radiographic images identified by CNN

The statistical results showed that CNN had different implant identification rates. Our study showed that the identification rate of CNN of the platform transfer department of Bicon was 74.5%, whereas that of repaired parts was 65.5%. Moreover, the identification rates of those two sites in the Straumann BL system were only 36.4% and 10.9%, respectively. This difference indicated that, after the training process, the CNN model identified a significant difference in the neck area between both implants and placed a high weight on that site during the training process.^18^ We propose two hypotheses to explain the possibility of differences in the identification of the implants’ neck area. First, the implants themselves are designed differently. Compared with the Straumann BL system, the Bicon has a sloping neck design at the platform transfer, resulting in significant narrowing,^31^ which may make the area a feature of the image extracted by deep learning. Second, we noticed that the areas covered by the thermal map of the implant contained not only the implant component, but also a portion of the bone component. Therefore, bone attachment around the implant may also affect the extraction of implant radiographic image features by the CNN.

However, the above two hypotheses were only proposed based on thermal maps and two‐dimensional measurement results, and there are still many limitations and uncertainties. The root cause of the identification differences may need further study in light of the principles and procedures of the CNN model.

**FIGURE 3:**
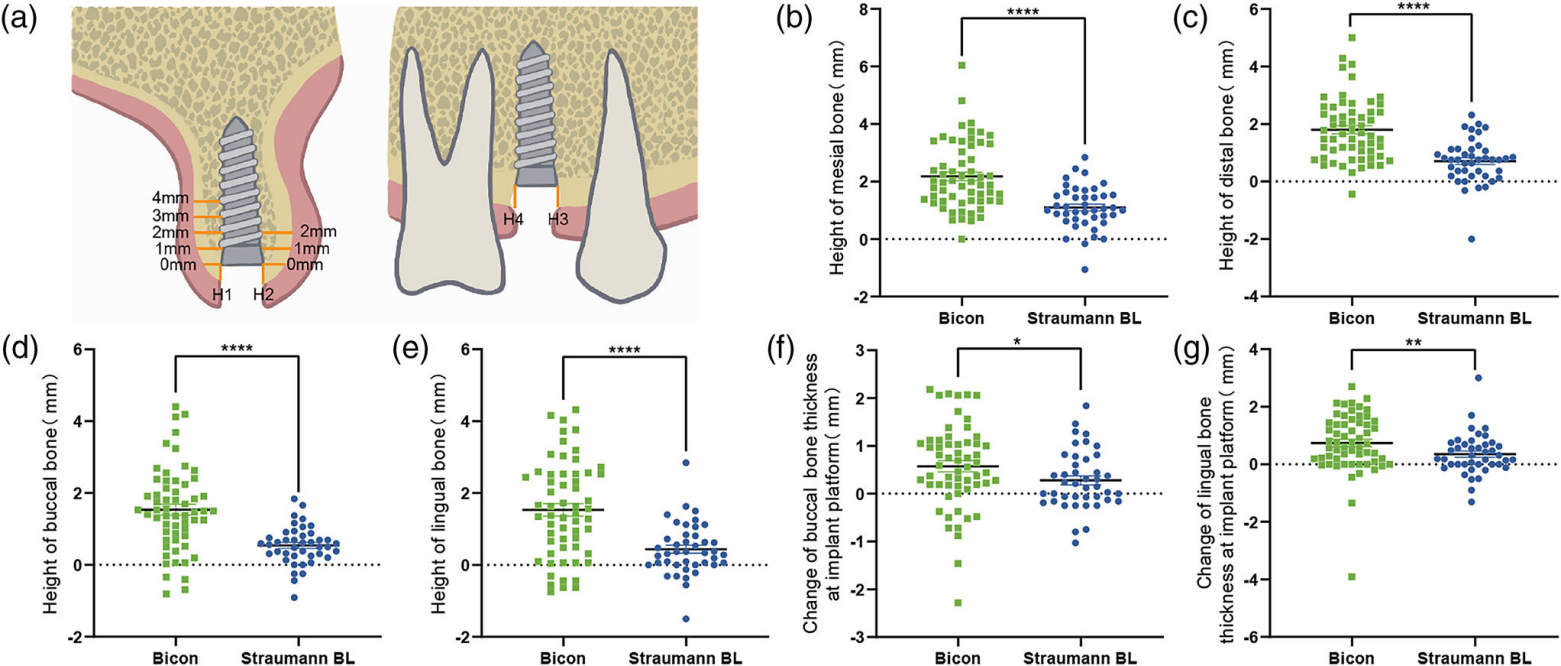
Statistical analysis results of peri‐implant bone measurement. Part a is the measurement diagram; parts b–e used the *t*‐test; and parts f and g used the rank sum test.

Using the Onevolume Viewer to measure 12 positions of 101 implants in 77 randomly selected patients. As shown in Figure 3b and c, the mesial and distal bone heights of the Bicon implants were 2.18 ± 1.17 mm and 1.80 ± 1.09 mm, whereas the mesial and distal bone heights of the Straumann BL implant were 1.09 ± 0.75 mm and 0.71 ± 0.77 mm. The mesial and distal bone heights of both implants were significantly different (*P* < 0.05). Since the two measurement sites of the mesial and distal bone heights are located in the implant neck area, there are differences in bone attachment between both implants’ neck, which may affect the extraction of image features by neural networks, thus leading to differences in identification sites.

### 3.3 Differences identified by CNN assists in studying the effect of implant type on clinical implantation outcomes

The observation of differences identified by CNN led us to focus on the differences in the bone attachment around the neck. Subsequently, we measured and analyzed them according to clinical experience.^32^, ^33^ The measurements showed that the mean height of the mesial, distal, buccal, and lingual bone of the Bicon implant was 1.76 ± 1.21 mm, whereas the mean bone height of the Straumann BL implant in these four positions was 0.70 ± 0.73 mm. The statistical analysis of measurements is shown in Figure 3b–e. The heights of the mesial, distal, buccal, and lingual bone of the Bicon implant post‐op were greater than those of Straumann BL (*P* < 0.05), indicating that the neck of Bicon implant was placed approximately 1 mm deeper than the Straumann BL implant. The amount of bone thickness variation at the buccal and lingual sides of the implant platform was 0.57 ± 0.89 mm and 0.74 ± 1.03 mm for the Bicon implants and 0.28 ± 0.61 mm and 0.35 ± 0.72 mm for Straumann BL implants at these two positions, respectively. The statistical analysis of measurements is shown in Figure 3f and g. The amount of bone thickness variation at the buccal and lingual sides of the Bicon implant platform was greater than that of the Straumann BL implant (*P* < 0.05), indicating that there was more bone resorption in the neck with Bicon implants between the first and second stages of surgery.

The findings mentioned above are derived from the CNN training process, which presents differences in implant neck identification rather than from the professionals' direct conjecture about how implant type affects implant depth and bone resorption. Clinical studies have shown that the implant placement depths may affect both hard and soft tissues around the implant,^34^, ^35^ resulting in different peri‐implant bone remodeling.^36^ Bone resorption has been used as an indicator to identify biological complications such as peri‐implantitis and subsequently to determine the success rate of the implant.^37^, ^38^ Therefore, these results allowed the evaluation of the likelihood of complications and long‐term survival rates of Bicon implants and Straumann BL implants. In summary, we used the differences identified during the training process of the CNN classification model to extract the influence of implant type on implantation outcomes and operation process.

It is feasible and convenient to use CNN to investigate the influence of various factors on clinical outcomes. Previous studies tended to use AI to classify implant images,^9^, ^39^, ^40^ with much attention being paid to the specific results presented by AI. However, our study focused on the training process, that is, on the differences identified and accumulated by the CNN while training and the reasons behind the formation of differences. In this study, CNN and Grad‐CAM were used to help identify and present differences in the implant radiographic images caused by pre‐treatment, surgical, and restorative factors, especially those that cannot be detected directly by researchers. This is a further application of the large number of implant images in clinical work, showing the features that are not easy to find directly and helping to find the potential value in implant images. It's also a significant complement to the potentially limited vision of researchers.^41^ In addition to our clinical knowledge, we can evaluate radiographic image identification differences and use them to guide the clinical diagnosis and treatment in oral implantology. For example, the same method can be used to present image differences due to factors such as implant placement angle and occlusal design to assist in investigating their impact on clinical implantation outcomes.

## 4 CONCLUSION

According to the differences between Bicon and Straumann BL implant radiographic images identified and presented by the training process of the CNN classification model, we found that the identified‐neck‐area of the Bicon implant was placed deeper than the Straumann BL implant, and there was more bone resorption on the buccal and lingual sides at the Bicon implant platform between the first and second stages of surgery. This study proved that using the CNN classification model can identify image differences caused by implant type, which assisted us in studying the influence of implant type on implantation outcomes. In summary, we construct a new method to evaluate radiographic image identification results based on artificial intelligence. The central idea of this method is to identify image differences, which can complement the limited vision of researchers. According to these identification differences, researchers can further discuss the clinical value contained in implant images, so that a large number of clinical implant image data can be further applied. The logic behind our design can be extended to various fields with a high demand for medical expertise.

In the future, studies that include other factors, especially those that include larger amounts of data, are needed to implement the extracted clinical guidance and determine the validity and creativity of the new method for researching more clinical factor influences.

## AUTHOR CONTRIBUTIONS

**Xinxu Huang**: Conceptualization, Methodology, Validation, Formal analysis, Investigation, Writing—Original Draft, Writing—Review & Editing, Visualization. **Xingyu Chen**: Conceptualization, Methodology, Software, Validation, Investigation, Writing—Original Draft, Writing—Review & Editing. **Xinnan Zhong**: Conceptualization, Writing—Original Draft, Writing—Review & Editing, Visualization. **Taoran Tian**: Conceptualization, Methodology, Software, Validation, Resources, Writing—Original Draft, Writing—Review & Editing, Supervision, Project administration, Funding acquisition.

## CONFLICT OF INTEREST STATEMENT

The authors declare no potential conflicts of interest with respect to the authorship and/or publication of this article.

## Data Availability

The data that support the findings of this study are available from the corresponding author upon reasonable request.

## References

[1] Chackartchi T , Romanos GE , Sculean A . Soft tissue‐related complications and management around dental implants. Periodontol 2000. 2019;81:124‐138. 31407443 10.1111/prd.12287

[2] Morton D , Gallucci G , Lin WS , et al. Group 2 ITI consensus report: prosthodontics and implant dentistry. Clin Oral Implants Res. 2018;29:215‐223. 10.1111/clr.13298 30328196

[3] Jung RE , Al‐Nawas B , Araujo M , et al. Group 1 ITI consensus report: the influence of implant length and design and medications on clinical and patient‐reported outcomes. Clin Oral Implants Res. 2018;29:69‐77. 30328189 10.1111/clr.13342

[4] Gao Z , Guo Y , Zhang J , Zeng T , Yang G . Hierarchical perception adversarial learning framework for compressed sensing MRI. IEEE T Med Imaging. 2023. 10.1109/TMI.2023.3240862 37022266

[5] Xu C , Xu L , Ohorodnyk P , Roth M , Chen B , Li S . Contrast agent‐free synthesis and segmentation of ischemic heart disease images using progressive sequential causal GANs. Med Image Anal. 2020;62:101668. 32276185 10.1016/j.media.2020.101668

[6] Shin HC , Roth HR , Gao M , et al. Deep convolutional neural networks for computer‐aided detection: CNN architectures, dataset characteristics and transfer learning. IEEE Trans Med Imaging. 2016;35:1285‐1298. 26886976 10.1109/TMI.2016.2528162 PMC4890616

[7] Sitaula C , Hossain MB . Attention‐based VGG‐16 model for COVID‐19 chest X‐ray image classification. Appl Intell (Dordr). 2021;51:2850‐2863. 34764568 10.1007/s10489-020-02055-x PMC7669488

[8] Zhu YC , Jin PF , Bao J , Jiang Q , Wang X . Thyroid ultrasound image classification using a convolutional neural network. Ann Transl Med. 2021;9:1526. 34790732 10.21037/atm-21-4328 PMC8576712

[9] Sukegawa S , Yoshii K , Hara T , et al. Deep neural networks for dental implant system classification. Biomolecules. 2020;10:984. 32630195 10.3390/biom10070984 PMC7407934

[10] Tan J , Gao Y , Liang Z , et al. 3D‐GLCM CNN: a 3‐dimensional gray‐level co‐occurrence matrix‐based CNN model for polyp classification via CT colonography. IEEE Trans Med Imaging. 2020;39:2013‐2024. 31899419 10.1109/TMI.2019.2963177 PMC7269812

[11] Yan T , Xu W , Lin J , et al. Combining multi‐dimensional convolutional neural network (CNN) with visualization method for detection of Aphis gossypii glover infection in cotton leaves using hyperspectral imaging. Front Plant Sci. 2021;12:604510. 33659014 10.3389/fpls.2021.604510 PMC7917247

[12] Yu K‐H , Beam AL , Kohane IS . Artificial intelligence in healthcare. Nat Biomed Eng. 2018;2:719‐731. 31015651 10.1038/s41551-018-0305-z

[13] Anwar SM , Majid M , Qayyum A , Awais M , Alnowami M , Khan MK . Medical image analysis using convolutional neural networks: a review. J Med Syst. 2018;42:226. 30298337 10.1007/s10916-018-1088-1

[14] Litjens G , Kooi T , Bejnordi BE , et al. A survey on deep learning in medical image analysis. Med Image Anal. 2017;42:60‐88. 28778026 10.1016/j.media.2017.07.005

[15] LeCun Y , Bengio Y , Hinton G . Deep learning. Nature. 2015;521:436‐444. 26017442 10.1038/nature14539

[16] Ye H , Hang J , Chen X , et al. An intelligent platform for ultrasound diagnosis of thyroid nodules. Sci Rep. 2020;10:13223. 32764673 10.1038/s41598-020-70159-y PMC7410841

[17] Höhn J , Krieghoff‐Henning E , Jutzi TB , et al. Combining CNN‐based histologic whole slide image analysis and patient data to improve skin cancer classification. Eur J Cancer. 2021;149:94‐101. 33838393 10.1016/j.ejca.2021.02.032

[18] Guan Q , Wang Y , Ping B , et al. Deep convolutional neural network VGG‐16 model for differential diagnosing of papillary thyroid carcinomas in cytological images: a pilot study. J Cancer. 2019;10:4876‐4882. 31598159 10.7150/jca.28769 PMC6775529

[19] Lopez‐Antequera M , Gomez‐Ojeda R , Petkov N , Gonzalez‐Jimenez J . Appearance‐invariant place recognition by discriminatively training a convolutional neural network. Pattern Recogn Lett. 2017;92:89‐95.

[20] Srivastava N , Hinton GE , Krizhevsky A , Sutskever I , Salakhutdinov R . Dropout: a simple way to prevent neural networks from overfitting. J Mach Learn Res. 2014;15:1929‐1958.

[21] Jeczmionek E , Kowalski PA . Flattening layer pruning in convolutional neural networks. Symmetry. 2021;13:1147.

[22] Mondal MRH , Bharati S , Podder P . CO‐IRv2: optimized InceptionResNetV2 for COVID‐19 detection from chest CT images. PLoS One. 2021;16:e0259179. 34710175 10.1371/journal.pone.0259179 PMC8553063

[23] Shamsi A , Asgharnezhad H , Jokandan SS , et al. An uncertainty‐aware transfer learning‐based framework for COVID‐19 diagnosis. IEEE Trans Neural Netw Learn Syst. 2021;32:1408‐1417. 33571095 10.1109/TNNLS.2021.3054306 PMC8544942

[24] Apostolopoulos ID , TBJa Image . Processing V. Covid‐19: automatic detection from X‐Ray images utilizing transfer learning with convolutional neural networks. Phys Eng Sci Med. 2020;43:635‐640. 32524445 10.1007/s13246-020-00865-4 PMC7118364

[25] Chattopadhay A , Sarkar A , Howlader P , Balasubramanian VN . 2018 IEEE winter conference on applications of computer vision (WACV). Grad‐CAM++: Generalized Gradient‐Based Visual Explanations for Deep Convolutional Networks. IEEE. Lake Tahoe; 2018:839‐847.

[26] Jiang B , Zhang Y , Zhang L , HdB G , Vliegenthart R , Xie X . Human‐recognizable CT image features of subsolid lung nodules associated with diagnosis and classification by convolutional neural networks. Eur Radiol. 2021;31:7303‐7315. 33847813 10.1007/s00330-021-07901-1

[27] Panwar H , Gupta PK , Siddiqui MK , Morales‐Menendez R , Bhardwaj P , Singh V . A deep learning and grad‐CAM based color visualization approach for fast detection of COVID‐19 cases using chest X‐ray and CT‐Scan images. Chaos Solitons Fractals. 2020;140:110190. 32836918 10.1016/j.chaos.2020.110190 PMC7413068

[28] Ye L . Current dental implant design and its clinical importance. Hua Xi Kou Qiang Yi Xue Za Zhi. 2017;35:18‐28. 28326723 10.7518/hxkq.2017.01.003 PMC7030203

[29] Zekry A , Wang R , Chau AC , Lang NP . Facial alveolar bone wall width—a cone‐beam computed tomography study in Asians. Clin Oral Implants Res. 2014;25:194‐206. 23294441 10.1111/clr.12096

[30] Slagter KW , Raghoebar GM , Vissink A , Meijer HJ . Inter‐ and intraobserver reproducibility of buccal bone measurements at dental implants with cone beam computed tomography in the esthetic region. Int J Implant Dent. 2015;1:8. 27747630 10.1186/s40729-015-0007-1 PMC5005615

[31] Markose J , Eshwar S , Srinivas S , Jain V . Clinical outcomes of ultrashort sloping shoulder implant design: a survival analysis. Clin Implant Dent Relat Res. 2018;20:646‐652. 29671933 10.1111/cid.12608

[32] Abrahamsson I , Berglundh T . Effects of different implant surfaces and designs on marginal bone‐level alterations: a review. Clin Oral Implants Res. 2009;20:207‐215. 19663966 10.1111/j.1600-0501.2009.01783.x

[33] Merheb J , Quirynen M , Teughels W . Critical buccal bone dimensions along implants. Periodontol 2000. 2014;66:97‐105. 25123763 10.1111/prd.12042

[34] Schwarz F , Mihatovic I , Golubovich V , Schär A , Sager M , Becker J . Impact of abutment microstructure and insertion depth on crestal bone changes at nonsubmerged titanium implants with platform switch. Clin Oral Implants Res. 2015;26:287‐292. 25175742 10.1111/clr.12478

[35] Becker K , Klitzsch I , Stauber M , Schwarz F . Three‐dimensional assessment of crestal bone levels at titanium implants with different abutment microstructures and insertion depths using micro‐computed tomography. Clin Oral Implants Res. 2017;28:671‐676. 27417919 10.1111/clr.12860

[36] Valles C , Rodríguez‐Ciurana X , Clementini M , Baglivo M , Paniagua B , Nart J . Influence of subcrestal implant placement compared with equicrestal position on the peri‐implant hard and soft tissues around platform‐switched implants: a systematic review and meta‐analysis. Clin Oral Investig. 2018;22:555‐570. 10.1007/s00784-017-2301-1 29313133

[37] Krennmair S , Weinländer M , Forstner T , Krennmair G , Stimmelmayr M . Factors affecting peri‐implant bone resorption in four Implant supported mandibular full‐arch restorations: a 3‐year prospective study. J Clin Periodontol. 2016;43:92‐101. 26445457 10.1111/jcpe.12469

[38] Renvert S , Persson GR , Pirih FQ , Camargo PM . Peri‐implant health, peri‐implant mucositis, and peri‐implantitis: case definitions and diagnostic considerations. J Periodontol. 2018;89:S304. 29926953 10.1002/JPER.17-0588

[39] Lee JH , Jeong SN . Efficacy of deep convolutional neural network algorithm for the identification and classification of dental implant systems, using panoramic and periapical radiographs: a pilot study. Medicine (Baltimore). 2020;99:e20787. 32590758 10.1097/MD.0000000000020787 PMC7328970

[40] Hadj Saïd M , Le Roux MK , Catherine JH , Lan R . Development of an artificial intelligence model to identify a dental implant from a radiograph. Int J Oral Maxillofac Implants. 2020;36:1077‐1082. 33270045 10.11607/jomi.8060

[41] Mundim MB , Dias DR , Costa RM , Leles CR , Azevedo‐Marques PM , Ribeiro‐Rotta RF . Intraoral radiographs texture analysis for dental implant planning. Comput Methods Programs Biomed. 2016;136:89‐96. 27686706 10.1016/j.cmpb.2016.08.012

